# The Overarching Influence of the Gut Microbiome on End-Organ Function: The Role of Live Probiotic Cultures

**DOI:** 10.3390/ph7090954

**Published:** 2014-09-19

**Authors:** Luis Vitetta, Rachel Manuel, Joyce Yusi Zhou, Anthony W. Linnane, Sean Hall, Samantha Coulson

**Affiliations:** 1 Medlab, 66 McCauley St, Alexandria, Sydney, NSW, Australia; 2 Sydney Medical School, The University of Sydney, Sydney, NSW, Australia

**Keywords:** probiotics, *Lactobacillus*, *Bifidobacteria*, gastrointestinal tract, commensal bacteria, brain, kidneys, skeletal muscle, adipose tissue, heart

## Abstract

At the time of birth, humans experience an induced pro-inflammatory beneficial event. The mediators of this encouraged activity, is a fleet of bacteria that assault all mucosal surfaces as well as the skin. Thus initiating effects that eventually provide the infant with immune tissue maturation. These effects occur beneath an emergent immune system surveillance and antigenic tolerance capability radar. Over time, continuous and regulated interactions with environmental as well as commensal microbial, viral, and other antigens lead to an adapted and maintained symbiotic state of tolerance, especially in the gastrointestinal tract (GIT) the organ site of the largest microbial biomass. However, the perplexing and much debated surprise has been that all microbes need not be targeted for destruction. The advent of sophisticated genomic techniques has led to microbiome studies that have begun to clarify the critical and important biochemical activities that commensal bacteria provide to ensure continued GIT homeostasis. Until recently, the GIT and its associated micro-biometabolome was a neglected factor in chronic disease development and end organ function. A systematic underestimation has been to undervalue the contribution of a persistent GIT dysbiotic (a gut barrier associated abnormality) state. Dysbiosis provides a plausible clue as to the origin of systemic metabolic disorders encountered in clinical practice that may explain the epidemic of chronic diseases. Here we further build a hypothesis that posits the role that subtle adverse responses by the GIT microbiome may have in chronic diseases. Environmentally/nutritionally/and gut derived triggers can maintain microbiome perturbations that drive an abnormal overload of dysbiosis. Live probiotic cultures with specific metabolic properties may assist the GIT microbiota and reduce the local metabolic dysfunctions. As such the effect may translate to a useful clinical treatment approach for patients diagnosed with a metabolic disease for end organs such as the kidney and liver. A profile emerges that shows that bacteria are diverse, abundant, and ubiquitous and have significantly influenced the evolution of the eukaryotic cell.

## 1. Introduction

The microbial communities that colonize the human GIT have been collectively referred to as the gut *microbiota*. The resident commensal cohort adapts to the local environmental/milieu conditions of the human host and establishes a complex ecosystem in which host–microbe, milieu–microbe and microbe–microbe interactions oversee the composition and dynamics of the GIT microbial and host cell community.

A recent analysis of gut microbial communities illustrates how the commensal community in the GIT, alter their make-up according to the milieu composition that is derived from different nutritional practices. The proposal has documented that there may be three predominant GIT microbial family types that predominate in the gut and have been designated as enterotypes. These include the *Bacteroides*, *Prevotella*, and *Ruminococcus*, families of bacteria [1]. A subsequent study that also investigated the association of dietary and environmental variables with the gut microbiota reported that the GIT microbiome was an entity with functional plasticity. In effect this flexibility is subject to environmental/nutritional signal(s) adaptation as evidenced by changed patterns of enterotype governance [2]. Wu and colleagues also reported, that the faecal communities clustered into enterotypes, and were distinguished primarily by the levels of *Bacteroides* and *Prevotella* that were present [1]. They concluded that the enterotypes were strongly associated with long-term diets, mainly protein and animal fat (*Bacteroides*) against carbohydrates (*Prevotella*). Furthermore, a controlled-feeding study of 10 subjects showed that microbiome compositions changed detectably within 24 h of initiating a high-fat/low-fiber or low-fat/high-fiber diet, whilst enterotype identity remained stable during the 10-day study. This data indicated that alternative GIT enterotype conditions could be dependent on long-term dietary patterns. Hence what is the biological significance of these studies, as yet, remains inconclusive [2]. However, enterpotype designation may not be as clear cut as envisaged, given that these human-associated bacterial diversity studies have categorized individuals into enterotypes/clusters based on the abundances of key bacterial genera in the gut microbiota. A recent meta-analysis of microbial community structures in humans recommends that multiple approaches may be required when testing and comparing for enterotypes [3].

As recent studies begin to report variations in gut metabolites our understanding of the host microbiome variations in health and disease progresses. For example it has been reported that in individuals with enriched gut microbe types (e.g., increased proportions of *Prevotella* in the gut exhibit a significantly higher plasma concentration of trimethylamine-*N*-oxide a pro-atherogenic metabolite) than individuals with a *Bacteroides* enterotype [4]. This very much indicating that enterotypes and their variations affect the host. Moreover Roager and colleagues [5] have recently shown that the ratio, of *Prevotella* spp to *Bacteroides* spp provides an additional stratification step that further fine tunes the profile of gut enterotypes. This may further enhance assessment of gut directed interventions in health and disease states.

Over the past several decades though, research has seen a refocusing of thinking and effort directed towards elucidating the critical inter-relationships that exist between the GIT microbiome and its host. This research has redefined the interactions between gut microbes and vertebrates, now recognising that the microbial active cohort and its mammalian host have shared co-evolutionary metabolic interactions that span millennia. Microbial interactions in the GIT provide the necessary cues for the development of regulated pro- and anti-inflammatory signals that promotes immunological tolerance, metabolic regulation and other factors which may then control local and extra-intestinal inflammation.

Furthermore, it is also becoming apparent that the GIT with its commensal cohort is a central regulator for the activities of end organs such as the kidneys, brain, adipose tissue, muscle and liver and as such may provide local prompts that are transmitted extra-intestinally to end organ sites. A scientific insight therefore has emerged that plausibly links the GIT with the physiology of end organ function that may influence health maintenance or trigger and support a disease state. Maintaining a healthy GIT milieu and epithelium with the administration of probiotics may constitute a novel therapeutic strategy for health.

## 2. Methods

A systematic search of the literature covering the years 2000–2014 was conducted using PubMed, the Cochrane Library, Science Direct, Scopus, EMBASE, MEDLINE and CINAHL.

### 2.1. Search Terms

**Figure 1:**
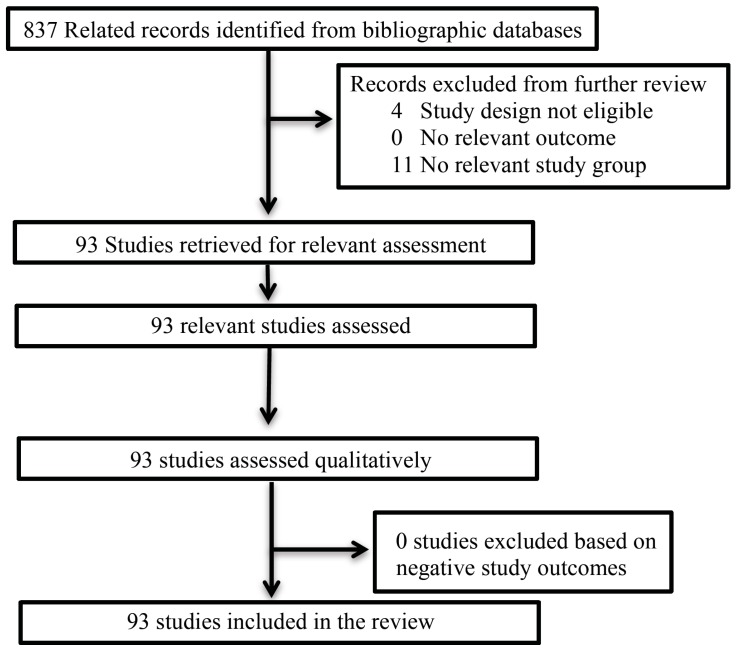
Flow diagram of studies for limited review.

Articles were identified using the search terms, “Probiotics” OR “Prebiotics” OR “Commensal Bacteria” OR symbiotics AND “Gastrointestinal Tract Diseases” and “Brain” AND “Kidney Disease” AND “Adipose Tissue” AND “Joint Diseases” AND “Liver Diseases” AND “Lung Diseases” AND “Immune Deficits”. The Inclusion criteria for this review were: (1) An RCT and/or cross-over clinical trial that used either a placebo comparator or other as a control; (2) Human participants (children, adolescents or adults); (3) The clinical study was published in English. A flow diagram of studies included and excluded is presented in Figure 1.

## 3. Clinical Studies

Probiotics are live bacterial cultures that are added to foods (e.g., yoghurts) and employed as dietary supplements, that when orally administered can improve the health of the host beyond their fundamental basic nutritional content [6]. Probiotic bacteria encompass those from different genera (as for example *Lactobacilli*, *Bifidobacterium*, *Escherichia*, *Saccharomyces* (a yeast), *Streptococcus*) giving rise to a variety of different species of each genera (*i.e.*, *Lactobacillus acidophilus*; *Lactobacillus bulgaricus*, *Lactobacillus rhamnosus*); that lead to different strains within a species (*i.e.*, *Lactobacillus acidophilus La-1*, *Lactobacillus acidophilus* NCFM). This taxonomic differentiation, importantly emphasizing that different strains from the same bacterial species may exhibit variable activity and as such may elaborate different physiological functions within the GIT [7] whilst exhibiting overlapping or specific therapeutic actions to different organ systems [8].

### 3.1. Probiotics and the GIT

**Figure 2:**
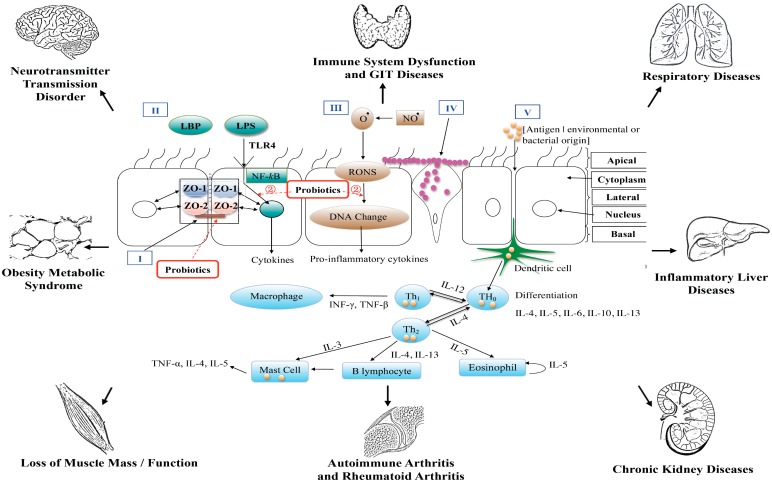
A diagrammatic representation of the epithelial barrier of the gastrointestinal tract and end–organ associations.

Perhaps the most studied site for investigating probiotic efficacy is the GIT. It has been reported [9] that probiotic bacteria may operate on three levels of host functionality that enhances GIT and extra intestinal functions (Figure 2) namely, (i) interfering with the growth of pathogenic bacteria in the lumen of the GIT; (ii) strengthening the epithelial gut lining’s barrier function and mucosal immunity as well as mucus production; and (iii) beyond the gut, have an effect on the systemic immune system, as well as other cell and organ systems such as the liver. Numerous studies have reported the efficacious use of probiotics (Table 1) [10,11,12,13,14,15,16,17,18,19,20,21,22,23,24,25,26,27,28,29,30,31,32,33,34,35,36,37,38,39,40,41,42,43,44]. Irritable bowel syndrome causes abdominal pain, bloating and alternating constipation and diarrhea. Clinical studies with probiotics overall have demonstrated efficacy for reducing abdominal pain [10,11,12,13,14,15,16,17,18]. In clinical trials [19,20,21] investigating functional abdominal pain and associated symptomatology two studies [19,20] that employed.

*L. reuteri* demonstrated efficacy. Several clinical studies have investigated the administration of probiotics in reducing antibiotic associated diarrhea [22,23,24,25,26,27,28,29]. Most studies demonstrated efficacy in reducing the development of antibiotic associated diarrhea and or duration of antibiotic associated diarrhea [23,24,26,27,28,29]. A number of clinical investigations have tested the efficacy of probiotics to reduce *helicobacter pylori* infection [30,31,32,33,34,35]. All studies included in this review bar one [30] reported an efficacious outcome with probiotic administration. Furthermore, several studies have also clinically investigated the efficacy of probiotics for functional gastrointestinal symptoms (e.g., pouchitis among others) [36,37,38,39,40,41]. All studies except two [39,41] reported an efficacious outcome. We reviewed three studies with very low weight or pretern birth infants and the efficacy of probiotics and the outcomes were contentious [42,43,44].

### 3.2. Probiotics and the Liver

A recent report has advanced the hypothesis that there exits a gut–liver axis that suggests the GIT microbiota may significantly affect liver physiology and act as a co–factor in the etiology of chronic liver disease [45]. This hypothesis has stemmed largely from the longstanding practice of using lactulose in the treatment of hepatic encephalopathy [46]. This then, suggesting gut microbiota involvement in the management of chronic liver disease. A GIT microbiota that sustains a persistent low level pro–inflammatory pathogenic profile could modulate liver damage caused by ethanol and other toxic compounds such as acetaldehyde, phenols and endotoxins.

Table 2 summarizes numerous studies that have employed probiotics in the treatment of chronic liver diseases reporting significant improvements [47,48,49,50,51,52,53,54,55,56,57]. Clinical studies that demonstrated efficacy were related to improving endotoxemia that in turn improved liver functionality [47,48,49,50]. It would seem that the probiotic actions most relevant to chronic liver diseases are modification of intestinal barrier function and the prevention of bacterial/toxin translocations. Increased GIT overloads with Gram-negative bacteria, increased permeability and impaired immunity may all contribute to increased bacterial/toxin translocations. Furthermore, a strong correlation between the rate of bacterial/toxin overload and the severity of cirrhosis was demonstrated [51,52,53,54,55]. Hence, multi-strain probiotics may alter gut flora and rescue the GIT microbiome towards a protective commensal bacteria profile with a concomitant increase in GIT epithelial barrier function.

### 3.3. Probiotics and Obesity

*In vitro* screening-experiments with bacteria from the genus *Lactobacillus* and *bifidobacteria* isolated from the human GIT have demonstrated significant cholesterol-lowering actions [58]. Recent findings suggest that a high-fat diet and the GIT bacterial cohort interact to promote early inflammatory changes in the gut that contribute to the development of obesity and insulin resistance [59].

Table 3 presents clinical studies that have investigated probiotic preparations in obesity [60,61,62,63,64]. The overall trend is that probiotic preparations could positively influence weight reduction. Specifically, in a study with healthy infants [60] it was demonstrated that probiotic administration significantly lowered levels of palmitoleic acid and significantly increased levels of putrescine. The data suggest that palmitoleic acid a major monounsaturated fatty acid (MUFA) that is strongly linked to visceral obesity was reduced with probiotic supplementation. While putrescine a polyamine with importance for gut integrity was beneficially increased. Probiotic supplementation in adulthood [61] and during the childhood (from birth to 10 years) [62] demonstrated that probiotics at least in part assisted with the control of abdominal visceral and subcutaneous fat. In an additional study administration of a multi-strain probiotic supplement provided a synergistic effect on overweight and obese individuals when provided with a weight loss diet [63]. In a further study with overweight children a multi-strain probiotic formulation significantly demonstrated decreased blood lipid profiles [64].

(I) Tight junction/gap junction between two epithelial cells. ZO translocates away from the cell boundary towards the nucleus reducing the trans-epithelial resistance allowing pathogens to move between the cells and through the cell wall into the *lamina propria*. Once the pathogen enters the *lamina propria* it is able to move throughout the gastrointestinal system and systemic circulation causing sever inflammation. (II) LPS mediated pathway of inflammation. (III) Dysregulated RONS mediated induction of cytokine production and inflammation. (IV) Mucus secreted from goblet cell forming a protective layer on the epithelial cell membrane. (V) Antigens presenting on the epithelial surface may be detected and consumed by dendritic cells. The antigen is presented to TH0 cells, which enter the Th1 or Th2 pathway depending on the antigen. Probiotics help regulate and reduce inflammation by (1) preventing the translocation of ZO to the nucleus helping maintain the TER and the integrity of gap junctions preventing the migration of pathogens past the epithelial barrier and (2) by preventing the activation of NF-κB caused by pathogens and compounds like LPS and RONS.

**Table 1:** Clinical studies of probiotics (with and without prebiotics) and gastrointestinal tract diseases.

Participant Type	Study Type (N° Patients)	Treatment	Duration	Results	Ref.
**Irritable Bowel Syndrome**					
Irritable bowel syndrome –constipation predominant	DBPCT (34)	1 × 10^8^ CFU/g *B. lactis* DN-173 010 125g/b.i.d.	4 weeks	↑Abdominal girth and gastrointestinal transit ↓Symptoms scores of IBS	[10]
Irritable bowel syndrome	DBPCT (55)	1 × 10^1^^0^ CFU/cap *L. rhamnosus* GG/b.i.d.	6 weeks	↓Abdominal pain	[11]
Irritable bowel syndrome	RCT (77)	1 × 10^1^^0^ CFU *B. infantis* 35624/o.i.d.	8 weeks	↓Abdominal pain Normalization of Th1/Th2 balance	[12]
Irritable bowel syndrome	DBPCT (40)	2 × 10^9^ CFU/mL *L. acidophilus*-SDC 2012/o.i.d.	4 weeks	↓Abdominal pain or discomfort	[13]
Irritable bowel syndrome	DBPCT (52)	2.5 × 10^1^^0^ CFU/cap *L. acidophilus* CUL60 *and* CUL21 *B. lactis* CUL34 *and B. bifidum* CUL20/o.i.d.	8 weeks	↓Symptoms scores of IBS ↑Scores for quality of life, days without pain and satisfaction with bowel habit	[14]
Irritable bowel syndrome	DBPCT (52)	5 × 10^7^ CFU/mL *L. paracasei ssp paracasei* F19 5 × 10^7^ CFU/mL* L. acidophilus* La5 5 × 10^7^ CFU/mL* B. lactis/*Bb12 200 mL/b.i.d.	8 weeks	No clear positive effect on IBS symptoms	[15]
Irritable bowel syndrome—diarrhea predominant	DBPCT (30)	1 × 10^8^ CFU/mL *S. thermophiles/*1 × 10^7^ CFU/mL *L. bulgaricus/* 1 × 10^7^ CFU/mL *L. acidophilus/*1 × 10^7^ CFU/mL *B. Longum* 200 mL/b.i.d.	4 weeks	↑IBS scores ↓Intestinal permeability	[16]
Irritable bowel syndrome	DBPCT(122)	1 × 10^9^ CFU/cap *B. bifidum* MIMBb75/o.i.d.	4 weeks	↑IBS scores	[17]
Irritable bowel syndrome –diarrhea predominant	DBPCT (297)	Inactivated *L. acidophilus* LB [dose administered not provided]	6 weeks	↓Number of stools	[18]
**Functional Abdominal Pain/Associated Symptoms**					
Functional gastroesphageal reflux	DBPCT(44)	1 × 10^8^ CFU/cap *L. reuteri* DSM 17938/o.i.d.	4 weeks	↓Median fasting antral area ↑Delta in gastric emptying rate ↓Median episodes per day of regurgitation	[19]
Functional abdominal pain	DBPCT (60)	2 × 10^8^ CFU/cap *L. reuteri* DSM 17938/b.i.d.	4 weeks	↓Abdominal pain	[20]
Functional gastrointestinal symptoms	DBPCT(17)	1-5 × 10^1^^0^ CFU *L. rhamnosus* GG/b.i.d	2 weeks	No evidence of efficacy	[21]
**Antibiotic Associated Diarrhea**					
Antibiotic-associated diarrhea	DBPCT (2941)	6 × 10^10^ CFU/cap *L.acidophilus* CUL60, and CUL21, *B. bifidum* CUL20, and *B lactis* CUL34/o.i.d	8 weeks	No evidence of efficacy	[22]
Antibiotic-associated diarrhea	DBPCT (89)	5 × 10^8^ CFU/g* L. acidophilus* Cl1285 and* L. casei* 98 g/o.i.d.	During antibiotic treatment	Prevention of antibiotic-associated diarrhea in hospitalized patients	[23]
Antibiotic-associated diarrhea	DBPCT (255)	50 or 100 × 10^9^ CFU/cap *L. acidophilus* CL1285 and *L. casei* LBC80R/o.i.d. or b.i.d. during antibiotic treatment	2 weeks	↓Risk of antibiotic-associated diarrhea	[24]
Antibiotic-associated diarrhea	DBPCT (275)	5 × 10^9^ CFU/cap *S. boulardii/b.i.d*	during treatment + 1 week	No preventing effect on the development of antibiotic-associated diarrhea	[25]
Antibiotic-associated diarrhea	DBPCT (437)	5 × 10^8^ CFU/g* L. acidophilus* CL1285^®^/*L. casei* 98 g/o.i.d. during antibiotic treatment	5 weeks	↓Duration of diarrhea	[26]
Antibiotic-associated diarrhea	DBPCT (113)	1 × 10^8^ CFU/mL* L. casei/L. bulgaricus/S. thermophilus* 97mL/b.i.d. during antibiotic treatment	2 weeks	↓Risk of antibiotic-associated diarrhea	[27]
Antibiotic-associated diarrhea	DBPCT (229)	4.5 × 10^11^ CFU/sachet *B. breve*, *B. longum*, *B. infantis*, *L. acidophilus*, *L. plantarum*, *L. paracasei*, *L.delbrueckii* subsp. *bulgaricus*, *S. thermophilus/b.i.d*	during antibiotic course+1 week	↓Risk of antibiotic-associated diarrhea	[28]
Acute rotavirus diarrhea	DBPCT (64)	4 × 10^1^^0^ CFU/dose *S. boulardii* or 6.625 × 10^7^ CFU/dose, *L.acidophilus,* 8.75 × 10^6^ CFU/dose *L. rhamnosus, B. longum and* 1.375 × 10^7^CFU/dose *S. Boulardii*/b.i.d	5 days	↓Median duration of diarrhea and fever in children who received the single species product ↓Vomiting in children who received the mixed species product	[29]
***Helicobacter pylori* eradication**					
*H. pylori* therapy	DBPCT (107)	1.25 × 10^9^ CFU *L. acidophilus,* 1.25 × 10^9^ CFU *L.rhamnosus,* 1.25 × 10^9^ CFU *B.bifidum and* *S. faecium* (b.i.d)	1 week	No evidence of increased efficacy	[30]
*H. pylori* therapy	Open label	30 × 10^8^ CFU *B. infantis/b.i.d*	2 weeks	↑ Cure rates	[31]
*H. pylori* therapy	DBPCT (88)	1 × 10^6^ CFU/g *L. acidophilus* LA-5/ 1 × 10^6^ CFU/g *B. lactis* BB-12 125 g/b.i.d. during *H. pylori* eradication	5 weeks	↓Duration of antibiotics-associated diarrhea ↓Gastrointestinal complaints	[32]
*H. pylori* therapy	Open label (228)	3 × 10^7^ CFU *L. acidophilus/o.i.d*	2 weeks	↑ Cure rates	[33]
*H. pylori* therapy	Open label (90)	1×10^8^ CFU *L.reuteri/o.i.d*	1 week	↑ Cure rates ↓Frequency and the intensity of antibiotic-associated side-effects	[34]
*H. pylori* positive subjects	DBPCT (22)	5 × 10^9^ CFU/tablet dead *L. reuteris* DSMZ17648/4 tablets/b.i.d	2 weeks	↓ *H. pylori*	[35]
**Functional gastrointestinal symptoms**					
Chronic pouchitis	DBPCT (20)	(0.5–1) × 10^10^ CFU/capsule *L. rhamnosus* GG/2 caps, b.i.d	12 weeks	↑Ratio of total faecal lactobacilli to total faecal anaerobes ↑Frequency of lactobacilli-positive cultures in the pouch and afferent limb mucosal biopsy	[36]
Functional gastrointestinal symptoms	TBPCT (87)	1.8 × 10^9^ or 17.2 × 10^9^ CFU/cap *B. lactis* HN019/o.i.d.	2 weeks	↓Whole gut transit time ↓Functional gastrointestinal symptoms	[37]
Healthy, postprandial intestinal gas-related symptom	DBPCT (61)	2 × 10^9^ CFU/cap *B. coagulans*/o.i.d.	4 weeks	↓Abdominal pain ↓Distension scoreNo significant differences in flatus, bloating and gas scores	[38]
Elderly patients receiving enteral feeding	DBPCT (123)	2.5 × 10^1^^0^ CFU/sachet *B. longum BB536* or 5 × 10^1^^0^CFU/sachet* B. longum BB536*/b.i.d	16 weeks	↑Bowel movements in patients with a low frequency of defecation ↓Bowel movements of patients with a high frequency of defecation	[39]
Elderly patients receiving enteral feeding	DBPCT (83)	5 × 10^1^^0^ CFU/sachet *B. longum BB536*/o.i.d	16 weeks	No significant changes in the frequency of defecation	[39]
Women with mild digestive symptoms	DBPCT (197)	1 × 10^8^ CFU/g *B. lactis* DN-173 010 125g/b.i.d.	4 weeks	↑Gastrointestinal well-being ↓Digestive symptoms	[40]
Women with minor digestive symptoms	DBPCT (324)	1. 10^7^ CFU/g *B. lactis* and 9.26 × 10^6^ CFU/g *S. thermophilus* and *L. bulgaricus*/125 g/b.i.d	4 weeks	No improvement in GI well-being	[41]
**Very low weight infants/preterm infants**					
Very low-birth weight infants	DBPCT (221)	3.5 × 10^18^ CFU/mL *L. sporogenes*	from first feed until discharge	No significant difference in the incidence of death or necrotizing enterocolitis ↓Feeding intolerance	[42]
Preterm infants	BRCT (81)	2 × 10^7^ CFU/g of milk powder *B. lactis* (daily milk volume increasing during treatment)	4 weeks	↓Intestinal permeability ↑Head growth	[43]
Prophylatic use in newborn infants	DBRCT (589)	*L reuteri* DSM 17938 Dose of 1 × 10^8^ CFU/day	12 weeks	↓the onset of functional gastrointestinal disorders	[44]

*L. = Lactobacillus; B. = Bifidobacteria*; *P. = Propionibacterium*; *S. = Saccharomyces (boulardii)*; *S. = Streptococcus (thermophilus)*; CFU: colony-forming unit; RCT: Randomized Controlled Trial; DBPCT: double bind placebo controlled trial; TBPCT: triple blind placebo controlled trial; o.i.d: once daily; b.i.d: twice daily; t.i.d: three times daily.

**Table 2:** Clinical studies of probiotics and liver disease.

Participant Type	Study Type (N°. Patients)	Treatment	Duration	Results	Ref.
Alcoholic liver disease	Open-label (66)	0.9 × 10^8^CFU/cap *B. bifidum*/0.9 × 10^9^CFU/cap *L. plantarum/*o.i.d.	5 days	Restoration of the bowel flora Improvement in alcohol-induced liver injury	[47]
Alcoholic liver disease	DBPCT (49)	2.5–25 × 10^9^CFU/cap *E. coli* Nissle twice the amount after 5 days/o.i.d.	6 weeks	Improvement of intestinal colonization in the *E. coli*, ↓Endotoxemia Improvement of liver functions	[48]
Alcoholic liver disease	Open-label (20)	6.5 × 10^9^CFU/cap * L. casei* Shirota*/*t.i.d.	4 weeks	Restore neutrophil function, * ex vivo* endotoxin- stimulated levels of sTNFR1, sTNFR2 and IL10 normalized TLR4 expression	[49]
Alcoholic liver disease, Nonalcoholic Fatty Liver Disease	Open-label (78)	4.5 × 10^11^CFU/cap *S. thermophilus/B. breve/B. longum/B. infantis/L. acidophilus/L. plantarum/L. casei/L. bulgaricus*/o.i.d.	12 weeks	Improvement of plasma level of MDA and 4-HE, whereas cytokines (TNF-alpha, IL-6, and IL-10) improved only in ALD patients	[50]
Cirrhosis	RCT (39)	*E. Nissle/*o.i.d. [dose administered not provided]	12 weeks	Improvement in intestinal colonisation Improvement in liver function assessed with the Child-Pugh classification.	[51]
Cirrhosis	RCT (81)	10^9^CFU/capsule *B. bifidus/L. acidophilus/L.bulgaricus* *S. thermophilus*/t.i.d.	2 weeks	↓*Escherichia coli* count ↓Intestinal flora imbalance Improvement in debilitation, food intake, appetite, abdominal distension, and ascitic fluid	[52]
Cirrhosis	DBPCT (36)	2 × 10^10 ^CFU/cap *L. acidophilus/L. bulgaricus/B. lactis/S. thermophilus*/o.i.d.	24 weeks	↓Ammonia levels starting after 1 month of treatment in patients with baseline ammonia levels > 50 mmol/LNo effect on liver enzyme	[53]
Cirrhosis	RCT (8)	1.8 × 10^12^CFU/cap *S. thermophiles/B. breve/B. longum/B. infantis/L. acidophilus/L. plantarum/L. casei/L. bulgaricus/*b.i.d.	8 weeks	↑Serum TNF-α ↓Plasma aldosterone.	[54]
Cirrhosis	RCT (50)	2.1 × 10^7^CFU/cap *Bifidobacterium/L. acidophilus/Enterococcus/*t.i.d. or 9 × 10^8^CFU/cap* B. subtilis/*1 × 10^8^ CFU/cap *E. faecium/*t.i.d.	2 weeks	↑ *Bifidobacterium* count ↓Fecal pH, fecal and blood ammonia. ↓Endotoxin in cirrhotic patients with endotoxemia (probiotics containing *Bacillus subtilis* and *Enterococcus faecium*)	[55]
Hepathic encephalopathy	DBPCT (55)	10^10^ CFU/cap *P. pentoseceus* 5–33:3/*L. mesenteroides* 32–77:1/*L. paracasei* subspecies *paracasei* 19/*L. plantarum* 2592/o.i.d.	4 weeks	↑Fecal content of non-urease-producing *Lactobacillus* species at the expense of these other bacterial species ↓Blood ammonia levels and reversal of HE ↓Endotoxemia.	[56]
Hepathic encephalopathy	DBPCT (60)	*B. longum/*o.i.d. And FOS [dose administered not provided]	12 weeks	Improving neuropsychological testing, serum ammonia levels	[57]

B. = *Bacillus E*. = *Enterococcus*; E. = *Escherichia coli*; P. = *Pediacoccus*.

Tien *et al.* [65] have reported that the anti-inflammatory effects of *Lactobacillus casei* are negatively associated with NF-κB activation. Figure 2 provides a diagrammatic view. Therefore, is has been hypothesised that health properties of probiotics could be related to peroxisome proliferator-activated receptor gamma (PPARg) activation, which then blocks the activity of NF-κB [66,67]. Hence it is interesting to note that over-consumption of food triggers GIT pro-inflammatory bacterial activity; this then may induce GIT metabolic dysfunction increasing the risk of metabolic diseases. Whereas a healthy diet with an optimally balanced GIT microbiota that promotes regulated/controlled PPARg activation could alleviate or suppress the risk of developing metabolic diseases such as T2DM.

### 3.4. Probiotics and the Brain

There is an increasing body of preclinical evidence that supports an important role that the gut microbiota may promote emotional behavior and may influence underlying brain mechanisms [68,69,70]. Studies with germ-free mice have demonstrated the important role of gut microbiota in brain development and resultant adult pain responses and emotional behaviors, as well as on adult hypothalamic-pituitary axis responsiveness.

Of the scant clinical trials that have investigated probiotics and brain behavior, the results have shown significant improvement in behavior with probiotic administration (Table 4) [71,72,73,74,75]. In one study assessing patients with traumatic brain injury, probiotic supplementation improved the anti-inflammatory clinical picture [74].

### 3.5. Probiotics and CKD

The dysfunction of the kidneys leads to disturbed renal metabolism and to impaired glomerular filtration and tubular secretion/reabsorption problems. This results in the retention of toxic solutes, which affect all organs of the body. It has been posited that toxins generated by gastrointestinal dysbiosis, and introduced into the body via the small and large bowel, may all contribute to CKD. They comprise advanced glycation end products, phenols and indoles [76]. Moreover, recent reports suggest that the bacterial load and the adverse products of the intestinal microbiota might influence chronic disease pathogenesis [1,2]. This is particularly relevant to the development of CKD, a disease of increasing prevalence in many Western societies. It has also been recently reported that the pharmacobiotic potential of the GIT micro–biometabolome may provide a plausible therapeutic role with the administration of live multi–strain probiotic cultures [77].

Although the current evidence as to the efficacy of probiotics to reduce uremic toxins is limited, the clinical evidence demonstrates that specific strains in a multiple–strain matrix configuration, in combination with prebiotics, may be most beneficial in reducing gut derived uremic toxins (Table 5) [78,79,80]. In addition, selecting probiotic species with known metabolic function, such as *Streptococcus thermophilus*, for metabolizing urea as a nitrogen growth source could contribute to reducing uremia.

**Table 3:** Clinical studies of probiotics and obesity.

Patients	Study Type (N° Patients)	Treatment	Duration	Results	Ref.
Healthy Infants	RCT (179)	1 × 10^8^ CFU/g *L. paracasei ssp. paracasei* F19/100 g b.i.d.	28 weeks	↓Palmitoleic acid ↑Putrescine	[60]
Adults with obese tendencies	DBPCT (87)	5 × 10^8^ CFU/g *L. gasseri* SBT2055 CFU*/*200 g daily	12 weeks	↓Abdominal visceral and subcutaneous fat areas ↓Body weight and other measures ↑High-molecular weight adiponectin in serum	[61]
Pregnant Women with obese tendencies	DBPCT (159)	1 × 10^1^^0^ CFU/cap *L. rhamnosus* GG*/*o.i.d.	4 weeks	Moderation of the initial phase of excessive weight gain of the children, but not of the second phase of excessive weight gain	[62]
Obese Adults	DBPCT (75)	1 × 10^8^ CFU/mL *L. acidophilus La5*/ 1 × 10^8^ CFU/mL* B. BB12/* 1 × 10^8^ CFU/mL *L. casei DN001/*o.i.d.	8 weeks	↓Expression of T-bet gene.	[63]
Overweight and obese children	TBPCT (70)	2.0 × 10^8^ CFU *L. casei, L.s rhamnosus, S. thermophilus, B. breve, L. acidophilus, B. longum* and *L. bulgaricus* with prebiotics (fructo oligosaccharides), Vitamin E, Vitamin A and Vitamin C/o.i.d	8 weeks	↓Serum triglycerides, total- and low density lipoprotein-cholesterol levels	[64]

SBCT: single bind controlled trial.

**Table 4:** Clinical studies of probiotics and brain disease.

Participant Type	Study Type (N° Patients)	Treatment	Duration	Results	Ref.
Anxiety-depressive symptoms	DBPCT (132)	10^8^ CFU/capsule *L. casei/*65mL/i.o.d.	3 weeks	Improvement in mood scores	[71]
Chronic fatigue syndrome	DBPCT (39)	8 × 10^7^ CFU/sachet *L. casei strain* Shirota/t.i.d.	8 weeks	↑Fecal total *Bifidobacteria* and *Lactobacillus* ↓Anxiety symptoms	[72]
Healthy adults	DBPCT (25)	3 × 10^9^CFU/sachet *L. helveticus* R0052*/*3 × 10^9^CFU/cap *B. longum* R0175/i.o.d.	2 weeks	↓Behaviors indicative of anxiety	[73]
Traumatic brain injury	SBCT (52)	0.5 × 10^8^CFU/sachet *B. longum/* 0.5 × 10^7^CFU/sachet *L. bulgaricus* 0.5 ×10^7^CFU/cap *S. thermophilues/*t.i.d.	3 weeks	Adjustment of the Th1/Th2 imbalance ↓Infection rate ↓Use of antibiotics ↑Level of IL-12	[74]
Healthy women with no gastrointestinal or psychiatric symptoms	DBPCT (36)	1.2×10^9^ CFU/cup *S. Thermophilues* *L. bulgaricus*/b.i.d.	4 weeks	↓Task-related response of a distributed functional network containing affective, viscerosensory, and somatosensory cortices	[75]

### 3.6. Probiotics and Joint Disease

Patients diagnosed with joint diseases have been reported as predisposed to GIT disturbances [81].

There are a small number of human clinical trials (Table 6) [82,83,84,85,86] that have assessed the therapeutic efficacy of administering probiotics to patients with autoimmune arthritic diseases. However, there are no clinical studies that have investigated the role of probiotics in reducing the symptoms of osteoarthritis. A recent animal study though has provided plausible data that a probiotic strain investigated, namely, *Lactobacillus casei* could act as a potent nutraceutical modulator for the treatment of osteoarthritis. Pain was reduced, as were inflammatory responses, and articular cartilage degradation [87].

### 3.7. Probiotics and Respiratory Diseases

Respiratory allergies include allergic rhinitis, sinusitis and asthma. The advent of the *hygiene hypothesis* has proposed that the increase in allergic diseases reflects a decrease in infections during childhood [88]. Clinical trials have also suggested that the exposure to microbes through the GIT robustly shapes immune function [89].

Probiotics have been reported to exert a beneficial effect in the prevention as well as the treatment of allergic diseases through modification of immune system of host via the GIT ecosystem. This has prompted studies (Table 7) [90,91,92,93,94,95,96,97,98,99,100,101] of feeding probiotics in prevention as well as the treatment of respiratory allergies. The clinical data presents a contentious profile of probiotic efficacy. In a recent controlled study it was reported that long–term consumption of fermented milk containing *Lactobacillus casei* may improve the health status of children with allergic rhinitis, however no effect was found in asthmatic children [92].

### 3.8. Probiotics and Skin Conditions

*Lactobacillus GG* has been reported to reduce clinical symptoms, intestinal inflammation and mucosal barrier permeability in infants with allergic dermatitis [102].

Allergic conditions are caused by abnormal or exaggerated immune reactions of the skin. A range of symptoms can be expressed however the most common chronic allergic conditions of the skin are atopic dermatitis/eczema. Probiotics are reported to exert some benefit in such conditions, which is thought to be due to the immune modulating effects of the bacteria. Studies demonstrate that probiotics contribute to relief of symptoms and also prevention of atopic conditions in infants (Table 8) [103,104,105,106,107,108,109,110,111,112,113,114,115,116,117,118,119]. In one study a probiotic preparation induced the repair of ultra violet damaged skin [103].

**Table 5:** Clinical studies of probiotics and chronic kidney disease.

Patients	Study Type (N° Patients)	Treatment	Duration	Results	Ref.
Chronic kidney disease (stages 3 and 4)	DBPCT (13)	1.5 × 10^9^ CFU/cap *L. acidophilus* KB31/*B. longum* KB35/*S. thermophilus* KB27/2 capsules/t.i.d.	24 weeks	Moderate changes in uric acid concentration No significant difference in serum creatinine concentration	[78]
Chronic kidney disease (stages 3 and 4)	DBPCT (246)	1.5 × 10^9^ CFU/cap *L. acidophilus/B. longum/S. thermophilus/*2 capsules/t.i.d.	24 weeks	↓Blood urea nitrogen. ↑Well-being with no serious adverse effects.	[79]
Chronic kidney disease	DBPCT (9)	1 × 10^8^ CFU *L. casei strain* Shirota/*B. breve strain* Yakult with 1.67 g galacto-oligosaccharides/t.i.d.	4 weeks	↑Quantity and normalization of the stools ↓Serum p-cresol level.	[80]

**Table 6:** Clinical studies of probiotics and joint diseases.

Patients	Study Type (N° Patients)	Treatment	Duration	Results	Ref.
Rheumatoid arthritis	PCT (21)	5 × 10^9^ CFU/cap *L. rhamnosus* GG*/*2 capsules/b.i.d.	52 weeks	No statistical significant differences in the activity of RA	[82]
					
Rheumatoid arthritis Sulfasalazine treated patients	PCT (12)	0.9 × 10^8^ CFU/sachet *L. acidophilus* L10*/B. lactis* B94*/S. salivarius* K12/b.i.d	12 weeks	No influence on the Sulfasalazine metabolism.	[83]
Rheumatoid arthritis	DBPCT (45)	2 × 10^9^ CFU/caplet *B. coagulans* GBI-30*/*b.i.d.	8 weeks	↓Pain scores. Improvement of global assessment and self-assessed disability	[84]
Rheumatoid arthritis	DBPCT (29)	2 × 10^9^ CFU/cap *L.s reuteri* RC-14/*L. rhamnosus* GR-1/b.i.d.	12 weeks	No differences observed	[85]
					
Spondyloarthritis	DBPCT (63)	1 × 10^8^ CFU/g * S. salivarius K12/*4 × 10^8^ CFU/g *B. lactis* LAFTI B94 1 × 10^8^ CFU/g *L. acidophilus* LAFTI L100.8 g/b.i.d.	3 weeks	No significant difference was noted between groups in any of the core domains	[86]

S. = *Streptococcus* (*salivarius*); PCT: Placebo Clinical Trial

**Table 7:** Clinical studies of probiotics and respiratory allergic diseases.

Participant Type	Study Type (N°. Patients)	Treatment	Duration	Results	Ref.
Asthma and allergic rhinitis	DBPCT (101)	2 × 10^9^ CFU/cap *L.gasseri*/o.i.d.	8 weeks	↓Clinical symptom scores ↓TNF-α, IFN-γ, IL-12, and IL-13 production by the PBMCs	[90]
Grass pollen-dependent allergic rhino-conjunctivitis	DBPCT (30)	2.5–25 × 10^9^ CFU/cap *E.coli Nissle* 1917/2 caps/o.i.d	24 weeks	No clinical evidence of efficacy	[91]
Allergic asthma and/or rhinitis	DBCT (187)	1 × 10^1^^0^ CFU/mL *L. casei/*100 mL/o.i.d.	52 weeks	No difference	[92]
Perennial allergic rhinitis	DBPCT (49)	3 × 10^8^ CFU/mL *L. acidophilus strain* L-92/100 mL/o.i.d.	8 weeks	No difference in IgE level or Th1/Th2	[93]
Seasonal allergic rhinitis	DBPCT (20)	1 × 10^5^ CFU/mL *L. casei* Shirota*/*65mL/o.i.d.	20 weeks	↓Antigen-induced IL-5, IL-6 and IFN- γ ↑IgG ↓IgE	[94]
High-risk allergy children	DBPCT (105)	5 × 10^9^ CFU/capsule *L.*GG/2 cap, o.i.d 6-4 weeks before delivery and 6 months after birth	30 weeks	No evidence of efficacy	[95]
High risk allergic disease infants	DBPCT (1223)	5 × 10^9^ CFU/cap *L. rhamnosus* GG*/*5 × 10^9^ CFU/capsule *L. reuteri* LC705 2 × 10^8^ CFU/cap *B. breve* Bb99*/*2 × 10^8^ CFU/cap *P. freudenreichii subspecies shermanii* JS/b.i.d [4 weeks before delivery + 24 weeks]	30 weeks	Protection from allergic disease only to cesarean-delivered children	[96]
Respiratory illness	DBPCT (523)	2.5 × 10^6^ CFU/mL *L.rhamnosus* GG (130 mL, t.i.c)	28 weeks	↓Occurence of respiratory illness	[97]
Japanese cedar pollinosis	DBPCT (44)	5 × 10^1^^0^ CFU *B. longum* BB536/b.i.d	13 weeks	↑ *Bacteroides fragilis* group	[98]
Infants	DBPCT (81)	1 × 10^9^ CFU/cap *L. rhamnosus* GG and 1 × 10^10^ CFU/cap *B. lactis* Bb-12/o.i.d	40 weeks	↓Risk of recurrent respiratory infections ↓ Acute otitis media ↓ Antibiotic use	[99]
Grass pollen-dependent allergic rhinitis	DBPCT (20)	2 × 10^9^ CFU/g *B. lactis* NCC2818/2g/o.i.d	8 weeks	↓Th-2 cytokines, secreted by stimulated blood lymphocytes ↓Total nasal symptom scores ↓Activated CD63 expressing basophils	[100]
Allergic rhinitis	DBPCT (31)	5 × 10^9^ CFU/mL *L. Helveticus* NCC1643 and × 10^7^ CFU/mL *L. paracasei ST11 /* 80 mL. o.i.d	4 weeks	↓Nasal congestion and nasal pruritus ↓IL-5, IL-8 and IL-10 secretion by peripheral blood mononuclear cells and serum allergen-specific IgG4	[101]

PBMC peripheral blood mononuclear cells; *P. freudenreichii*: *Propionibacterium freudenreichii*.

**Table 8:** Clinical studies of probiotics and skin conditions/diseases.

Participant Type	Study Type (N°. Patients)	Treatment	Duration	Results	Ref.
**UV induced skin damage**					
Ultraviolet-induced skin damage	CT (139)	5 × 10^8^ CFU *L. johnsoni*/before UVR exposure	3–6 weeks	Prevention the UV-induced decrease in Langerhans cell density ↑Factor XIIIa+ type I dermal dendrocytes ↓Dermal inflammatory cells ↑Minimal erythemal dose ↑ΔE* parameter	[103]
**Pregnant women carrying high risk allergy babies**					
High-risk allergy children	DBPCT (159)	1 × 10^1^^0^ CFU/cap* L. rhamnosus* strain GG/o.i.d. or b.i.d./3 weeks before delivery + 24 weeks	4 weeks to mothers and 24 weeks to infants	↓cumulative risk for developing eczema during the first 7 years of life	[104]
Pregnant women carrying high-risk allergy children	DBPCT (1223) mothers (925) infants	5 × 10^9^ CFU/cap *L. rhamnosus* GG 53103/5 × 10^9^ CFU/cap*/L. rhamnosus* LC705 7061 CFU/cap/5 × 10^9^ CFU/cap* B. breve* Bb99 13692 and 2 × 10^8^ CFU/cap *P. freudenreichii* ssp. *shermanii* JS 7076/b.i.d.+ GOS daily	Mothers dosed with multi strain probiotics for 2 to 4 weeks before delivery then infants received probiotics +GOS for 24 weeks	Prevention of eczema at 2 years of age ↑*Lactobacilli* and *Bifidobacteria* in the gut. No effect on incidence of allergic diseases. ↑ CRP, IgA, IgE, IL-10 which were associated with ↓ risk of eczema.	[105,106]
Pre and post natal probiotic supplementation	DBPCT (61)	*L. reuteri* 1 × 10^8^ CFU/day to mothers from week 36 of pregnancy and then to the infant for 24 months post delivery.	52 weeks	↓IgE-associated eczema and lowered allergen and mitogen responsiveness	[107]
Maternal probiotic supplementation during pregnancy	DBPCT (205)	*L. rhamnosus* LPR (CGMCC 1.3724) and *B. longum* BL999 (ATCC: BAA-999) or the combination ST11 and BL999 (ST11 BL999) consisting of *L. paracasei* ST11 (CNCM 1–2116) and *B longum* BL999. Dose 1 × 10^9^ CFU/day provided in 1 sachet of 7 g/d (powder form) which was diluted in a glass of water.	8 weeks	↓risk of eczema in infants with allergic mothers positive for skin prick test.	[108]
**Atopic dermatitis/eczema with/without cow’s milk/food allergies**					
Atopic dermatitis	DBPCT (90)	5 × 10^9^ CFU/g *L. acidophilus* DDS-1/*B. lactis* UABLA-12/1g, b.i.d	8 weeks	↓SCORAD* score ↓CD4 and CD25 lymphocytes ↑CD8 Lymphocytes	[109]
High-risk atopic dermatitis children	PCT (15)	*B. breve* M-16V strain [dose administered not provided]	4 weeks	↑Proportion of *Bifidobacteria* in the fecal microflora ↓Proportion of aerobic bacteria ↓allergic symptoms	[110]
High-risk allergy children	DBPCT (132)	0.5 × 10^6^ CFU/cap LGG/2 capsules/o.i.d.	28 weeks	Preventive effect on the incidence of eczema in high-risk children	[111]
High risk atopic eczema children	DBPCT (132)	0.5 × 10^6^ CFU/cap *L. rhamnosus* 53103 2 caps/o.i.d.	28 weeks	Preventive effect on the incidence of eczema in high-risk children	[112]
Atopic dermatitis	DBPCT (58)	1 × 10^6^ CFU *B. bifidum* BGN4/1 × 10^6^ CFU *B. lactis* AD011/1 × 10^6^ CFU *L. acidophilus* AD031/o.i.d.	32 weeks	↓Cumulative incidence of eczema no difference in serum total IgE level or the sensitization against food allergens	[113]
High-risk atopic dermatitis children	DBPCT (102)	1 × 10^9^ CFU/sachet *B. bifidum*/1 × 10^9^ CFU/sachet *B. lactis*/1 × 10^9^ CFU/sachet *L. lactis*/o.i.d. 8 weeks before delivery + 58 weeks.	Prenatal administration to mothers and for 52 weeks to infants post birth	Preventive effect on the incidence of eczema in high-risk children	[114]
Atopic dermatitis	DBPCT (59)	2 × 10^10^ CFU/g *L. rhamnosus* and *B. Lactis* [dose administered not provided]	4 weeks	↓SCORAD geometric mean score	[115]
Atopic eczema/dermatitis syndrome and food allergy	DBPCT (230)	5 × 10^9^ CFU/cap *L. rhamnosus* GG 53103 or 5 × 10^9^ CFU/cap L. rhamnosus GG*/*5 × 10^9^ CFU *L. rhamnosus LC705*/2 × 10^8^ CFU/cap*/B. breve* Bbi99 and 2 × 10^9^ CFU/capsule *P. freudenreichii ssp. Shermanii* JS/b.i.d.	4 weeks	↑Fecal IgA ↓Fecal alpha_1_-antitrypsin	[116]
Atopic dermatitis	DBPCT (66)	1 × 10^9^/sachet *L. fermentum* VRI-033 PCC (b.i.d)	8 weeks	↓SCORAD total scores	[117]
High-risk allergy children	DBPCT (425)	6 × 10^9^ CFU/day *L. rhamnosus* HN001 or 9 × 10^9^ CFU/day *B.animalis* subsp *lactis* HN019/from 35 weeks gestation to 2 years after birth	109 weeks	Protective effect of HN001 against eczema, when given for the first 2 years of life only, extended to at least 4 years of age. Protective effect against rhino-conjunctivitis	[118]
High-risk allergy children	DBPCT (474)	6 × 10^9^ CFU/cap *L. rhamnosus*/3 weeks before delivery + 2 years	119 weeks	↓Cumulative prevalence of eczema No effect on atopy	[119]

* SCORAD = SCORing Atopic Dermatitis; UVR: Ultraviolet Radiation; UV-DL: Ultraviolet

## 4. Discussion

A diverse series of clinical trials implementing an assortment of probiotic preparations have frequently demonstrated efficacy, when investigating their administration effects on various end organ tissues (Table 1, Table 2, Table 3, Table 4, Table 5, Table 6, Table 7 and Table 8). The central theme of this activity posits that the GIT can influence numerous end organ tissues beneficially. Further, the clinical studies indicate that the administration of probiotics may provide efficacy in restoring the GIT microbiome to a more balanced metabolic state. This possibly achieved by partly controlling the pathogenic bacterial cohort that in turn beneficially affects end organ physiology.

Hence in this review/commentary we have advanced the hypothesis that a dysbiotic GIT that is induced by a microbiome drift toward an over–growth of pathogenic bacteria may play a significant role in the induction of pro–inflammatory mediators that begin in the GIT and then may affect different end organs as shown in Figure 2. The disruption of the GIT epithelial barrier that can accompany chronic use of analgesic medications (e.g., NSAIDs) exacerbating local pro–inflammatory responses induced by the pathogenic commensal cohort is such an example. This activity can further disrupt GIT physiological and epithelial barrier function leading to disruption of controlled pro-inflammatory actions.

The gut mucosa is the largest and most dynamic immunological environment of the body. It's often the first point of pathogen/antigen exposure and many microbes use it as a base position entry into the rest of the body. The gut immune system therefore needs to be prepared to respond to pathogens while at the same time it is constantly exposed to innocuous environmental antigens, food particles and commensal pathogens and their respective metabolites, which need to be tolerated. Misdirected immune responses to harmless antigens are the underlying cause of food allergies and debilitating conditions such as inflammatory bowel diseases. GIT dysbiosis describes bacterial imbalances usually in the GIT. Such imbalances may increase the risk of developing GIT barrier dysfunction, via enterocyte hyper–permeability [*leaky gut*] to bacterial endotoxins or environmental antigens.

The published research data recommends that minimum doses required to elicit a therapeutic benefit is strain dependent. Shornikova and colleagues have reported that 10^7^ bacteria of *L. reuteri* MM53 is sufficient to produce a beneficial effect [120]. However with other bacterial strains such as *L. rhamnosus* GG (lyophilised) 10^9^ viable bacteria is a requisite dose [121].

Research studies have produced conflicting evidence, with some studies demonstrating a therapeutic benefit with doses of 10^7^–10^8^ CFU/dose [121]. Clinical research trials that have reported efficacious outcomes administered probiotic strains with ≥ 10^9^ CFU/dose [120,122,123,124].

At present the best practice is to ensure that supplements contain strains with a concentration of 10^9^ CFU/dose or higher unless research demonstrates conclusively that efficacy is achieved at lower doses. Also, it seems that multi–strain probiotics favor enhanced efficacy over single strains. In preparations with multiple strains a similar strain concentration should apply.

What is becoming increasingly clear is that the pharmacobiotic nature of probiotic strains in the form of nutritional and functional food additives to regulate the gut microbiome is an exciting growth area of therapeutics, developing alongside an increased scientific understanding of gut–microbiome symbiosis in health and disease.

Although, readdressing the broad definition accustomed to probiotics may be difficult given that different strains have been shown to ameliorate similar symptoms in different end organs, the published clinical studies show that probiotics may have *drug like* effects. Hence therefore as such there is a need to further define probiotics at the strain level according to specific activities demonstrated and the robustness of that effect. An effort that is both convoluted and intellectually challenging.

## 5. Future Prospective

A growing number of studies have shown a correlation between dysbiosis of the gastrointestinal microbiome and end-organ disease. With the transient modulatory effects that probiotics can induce on the gastrointestinal microbiotia, there emerges a significant potential to counterbalance gastrointestinal dysbiosis for health restoration. During this last decade, the efficacy of probiotic supplementation has been studied in number of human diseases, including numerous conditions as for example irritable bowel syndrome, inflammatory bowel diseases, obesity and numerous allergic diseases (Table 1, Table 2, Table 3, Table 4, Table 5, Table 6, Table 7 and Table 8). Variations in probiotics species and strains used for clinical trials may be the primary reason for the variable effects that have been observed. This then serving to teach, that importantly standardized methods are required for the study of the gastrointestinal microbiome that, will allow valid comparisons from different groups to be made.

Modulation of the gut microbiota is one of the potential health-beneficial effects of probiotics. They have the capacity to modulate the intestinal microbiota by diverse mechanisms that include reduction of the luminal pH, competition for nutrients, secretion of anti microbial compounds or even prevention of pathogenic bacterial adhesion. Recent literature provides evidence that probiotics have immune-modulatory and anti-inflammatory effects. However, these effects can be strain-specific and species-dependant, thus knowing the physiological and the molecular mechanisms of each probiotic strain is an essential requisite for efficiently treating immune-mediated diseases. Thus contributing to the development of multi-strain probiotic formulas designed for specific interventions. This approach is paramount for testing probiotic efficacy otherwise the evidence will remain largely empirical, and clinical trial outcomes will vary and the potential of probiotics in disease treatments will remain obscure.

Furthermore, a common “hype” leveled at probiotics is that that they will cure all of disease. This posit is unequivocally disputed and not endorsed. An enhanced understanding of the functional GIT bacterial cohort that tolerates the host *versus* the pathogenic cohort that adversely affects the host will elucidate important relationships that exist between the indigenous microbiome and the human host. Probiotic preparations with specific metabolic properties (e.g., those strains that may increase mucus secretion) may provide clues for the future direction of clinical research into probiotics that confirm specific actions and doses to be administered for specific conditions. Hence the intention would then be to administer specific probiotic strains that beneficially modify the microbiome, albeit transiently, with specific beneficial actions directed at preventing or treating specific conditions (e.g., antibiotic associated diarrhea). Such research will lead to the further wide acceptance of live bacterial cultures as pharmacobiotic therapeutics.
